# Not Playing by the Rules: Exploratory Play, Rational Action, and Efficient Search

**DOI:** 10.1162/opmi_a_00076

**Published:** 2023-06-15

**Authors:** Junyi Chu, Laura E. Schulz

**Affiliations:** Department of Brain and Cognitive Sciences, Massachusetts Institute of Technology, Cambridge, MA

**Keywords:** play, exploration, rational action, preschoolers

## Abstract

Recent studies suggest children’s exploratory play is consistent with formal accounts of rational learning. Here we focus on the tension between this view and a nearly ubiquitous feature of human play: In play, people subvert normal utility functions, incurring seemingly unnecessary costs to achieve arbitrary rewards. We show that four-and-five-year-old children not only infer playful behavior from observed violations of rational action (Experiment 1), but themselves take on unnecessary costs during both retrieval (Experiment 2) and search (Experiments 3A–B) tasks, despite acting efficiently in non-playful, instrumental contexts. We discuss the value of such apparently utility-violating behavior and why it might serve learning in the long run.

## INTRODUCTION

Play is one of the most charming—and perplexing—behaviors of early childhood (see Berlyne, 1969; Bruner et al., 1976; Chu & Schulz, 2020; Doebel & Lillard, 2021; Lillard, 2015, 2017; Lockman & Tamis-LeMonda, 2021; Pellegrini et al., 2007; Scarlett et al., 2004; Singer et al., 2009; Zosh et al., 2018 for discussion and reviews). Play in humans and other animals may serve many biological and social functions, including acting as an honest signal of fitness (Alessandri, 1991; Sharpe et al., 2002; but see Held & Špinka, 2011), directly promoting fitness (Byers & Walker, 1995; Pellegrini & Smith, 1998), and supporting emotion regulation and promoting social bonds (Drea et al., 1996; Galyer & Evans, 2001; Gilpin et al., 2015; Lillard, 2017; Palagi, 2008; Panksepp, 1981). Some kinds of play may also be evolutionary spandrels (Gould & Lewontin, 1979), persisting simply because behaviors that are adaptive in some contexts tend to be repeated even in non-functional contexts. (Thus, dolphins who blow bubble nets to catch fish may continue to blow bubbles when no fish are around; McCowan et al., 2000; Pace, 2000; Sharpe & Dill, 1997). These different accounts of play are not mutually exclusive, and each likely characterizes some aspects of play behaviors across species (see Chu & Schulz, 2020 for review). However, our primary interest here is in cognitive accounts of play, and in particular the connection between exploratory play and learning.

A broad interest in the relationship between play and learning has influenced work in developmental psychology, education, and ethology for over a century (Groos, 1901; Gulick, 1920; Montessori, 1912) and today inspires both approaches to engineering autonomous agents in robotics, machine learning, and AI (Baranes & Oudeyer, 2010; Burda et al., 2018; Forestier et al., 2022; Pathak et al., 2017; see Colas et al., 2020; Oudeyer, 2018 for discussion) and a rich tradition in developmental cognitive science (e.g., Bonawitz et al., 2012; Lapidow & Walker, 2020; Legare, 2012; Perez & Feigenson, 2022; Siegel et al., 2021; Sobel et al., 2022; Stahl & Feigenson, 2015, 2018). We are advocates and contributors to this line of work and review it in detail to follow. However, play and learning are both complex phenomena and the relationship between the two remains far from simple. Here we focus on a fundamental challenge in connecting play to formal accounts of learning. We suggest that even in the context of relatively straightforward exploratory play, children tend to violate principles of efficient planning and rational action.

To begin however, we note that there is an abundant literature suggesting that children’s spontaneous exploration and exploratory play is indeed sensitive to opportunities for expected information gain. Indeed, the fact that infants look longer at events that violate their expectations has been the basis for infant looking time paradigms for many decades (see e.g., Csibra et al., 2016 for review) and recent work has attempted to quantify the information-theoretic factors that affect infants’ visual search (e.g., Kidd et al., 2012; Raz & Saxe, 2020; Sim & Xu, 2019).

Moreover, infants do not simply engage in rational visual search; they also selectively explore and manipulate objects in ways that are sensitive to uncertainty, prediction error and opportunities for information gain. Thus, for instance, infants selectively explore objects that appear to violate their prior expectations and intuitive theories. Thirteen-month-olds spend more time touching and reaching into a box that generated an unexpected sample (i.e., a uniform sample of colored balls from a box containing balls of many colors) than a box that generated an expected sample (i.e., balls of many colors; Sim & Xu, 2017b). Similarly, eleven-month-olds selectively bang objects if they appear to violate solidity but selectively drop them if they appear to violate gravity (Stahl & Feigenson, 2015, 2018). Critically, if infants are given information that explains away the seeming violations (e.g., the solid wall that the ball seemed to pass through is revealed to be an archway with a hole in it), they no longer engage in this kind of exploration (Perez & Feigenson, 2022).

The evidence for rational exploration is even more extensive in older children. Two to five-year-olds selectively choose, design, and communicate informative interventions that disambiguate evidence in play, exploring more when evidence violates their prior beliefs (e.g., Bonawitz et al., 2012; Legare, 2012; Schulz et al., 2008) and also when evidence is ambiguous or confounded (Schulz & Bonawitz, 2007; Sodian et al., 1991; van Schijndel et al., 2015). Moreover, children systematically explore longer in contexts where there is higher uncertainty (Lapidow et al., 2022; Siegel et al., 2021) and selectively explore in ways likely to generate informative evidence for themselves and others (Butler, 2020; Butler & Markman, 2012; Cook et al., 2011; Gweon & Schulz, 2018; Lapidow & Walker, 2020). Children also learn from the evidence they generate in play (Lapidow et al., 2022; Lapidow & Walker, 2020; Sim & Xu, 2017a; Sobel et al., 2022; Walker & Gopnik, 2014).

Such findings about exploratory play are broadly consistent with work on children’s early understanding of principles of rational action. Even infants expect agents to act efficiently to achieve their goals (e.g., Csibra et al., 2003; Gergely & Csibra, 2003; Gergely et al., 1995; Liu et al., 2017; Liu & Spelke, 2017; Mascaro & Csibra, 2022; Varga et al., 2021), and toddlers and preschoolers engage in rational planning, weighing the cost of acting against its value (Bridgers et al., 2020; Liu et al., 2019; Sommerville et al., 2018) and assuming others will do the same (Aboody et al., 2021; Jara-Ettinger et al., 2015, 2017).

However, the focus on identifying aspects of exploratory play consistent with accounts of rational exploration arguably obscures some of the richness of play behavior itself. This becomes clear in considering the gap between any laboratory experiment on play and what children might do with the same stimuli if left to their own devices. Thus, for instance, four to eight-year-olds may well shake a box of marbles longer in proportion to how difficult it is to distinguish competing hypotheses about its contents (Siegel et al., 2021). However, on their own, children might never generate that specific behavior and would instead presumably engage in all manner of behaviors the details of which would be hard to predict a priori (e.g., rolling the marbles across the table, arranging them into patterns, or placing the box on top of the marbles to make a toy car). Indeed, arguably, children’s play may be characterized by nothing so much as their tendency to invent novel goals and problems for themselves (Chu & Schulz, 2020).

The idiosyncratic nature of children’s play has led some researchers to emphasize not the efficiency or rationality of play but its seeming arbitrariness, leading to proposals that the randomness and variability associated with play may themselves be important to learning (e.g., Dayan & Sejnowski, 1996; Gordon, 2020; Ossmy et al., 2018). However, neither random behavior, nor a mere preference for doing new things, is likely to support learning in open-ended contexts where rewards are sparse (Oudeyer et al., 2007). Moreover, children at play do not simply engage in random behaviors (Meder et al., 2021); they invent novel goals and plans (“Let’s go down the slide backwards”; “Let’s cross the dining room without touching the floor”; e.g., Colliver & Fleer, 2016).

We are struck by children’s ability to invent new problems, goals, and constraints for themselves, and, in particular, by children’s willingness to incur seemingly unnecessary costs to achieve arbitrary rewards. One might account for some kinds of costly, self-handicapping behaviors (e.g., crows and primates deliberately balancing on unstable branches) with respect to motor learning and adaptive skill building (Petrů et al., 2009; Spinka et al., 2001). However, it is less clear what to make of the full range of seemingly unnecessary costs children incur in play, especially when children have already acquired the requisite motor skills (“Let’s crawl around under the bed sheet”; “Let’s stick the crayons into vent”; “Let’s blow the peas off the plate”). Moreover, the gap between the complexity of play behaviors and its payoff in generalizable skills only becomes greater as play becomes more elaborate (“Let’s use black and white stones to control territories on a grid”). Given the richness, variability, and arbitrariness of the problems humans create in play, we are disposed to take the arbitrariness of much distinctively human play seriously, and to consider the value that taking on new (and prima facie unnecessary) costs might have for the flexibility and productivity of human cognition.

The first step—and the focus of the current paper—is to look at whether children’s exploratory play is indeed distinguished from functional behavior by the prevalence of seeming violations of rational, efficient action. Although many games are characterized by otherwise unnecessarily costly actions (e.g., jumping over chalk lines in hopscotch; picking up objects before the next bounce of a ball in jacks) to our knowledge, no previous work has investigated this phenomenon experimentally. Here we look both at whether children use apparent violations of principles of rational action to decide when others are playing (Experiment 1) and whether children themselves adopt unnecessary costs when playing. To establish the generality of the phenomena, we look at both retrieval tasks (Experiment 2) and exploration tasks (Experiment 3).

We focus on preschoolers on pragmatic grounds: They are the youngest children we can test with the linguistic and executive function skills to follow simple task instructions. Although we rely on explicit verbal instructions (to play or to achieve a functional goal), our tasks do not require or assume that children have metacognitive awareness of their tendency to incur unnecessary costs in play (see Goodhall & Atkinson, 2019; Wing, 1995 for work on children’s metacognition about other aspects of play.). Much as children recognize and produce grammatical sentences without knowing how, children might adopt different costs and rewards in play and expect others to do the same without explicit awareness. Note also that the current study is designed to establish whether children selectively engage in inefficient actions during play. Because it is not yet clear to what extent the phenomenon exists, we will focus here on characterizing the behavior and leave for future work any consideration of mechanisms that might underlie any observed developmental changes across ages.

## EXPERIMENT 1

Our first question is whether violations of rational action might help children recognize others’ behavior as playful. Of course, play is not the only possible explanation for seeming violations of rational action. Many valuable behaviors (social norms and rituals, steps necessary for effective tool use, safety procedures, etc.) are cognitively opaque (see e.g., Kenward et al., 2011; Keupp et al., 2013, 2018; Rakoczy et al., 2008) and researchers have suggested that children’s tendency to imitate even seemingly unnecessary, inefficient actions may be critical for transmitting both instrumental skills and social conventions (Horner & Whiten, 2005; Keupp et al., 2018; Legare & Nielsen, 2015; Legare et al., 2015; Lyons et al., 2007; Nielsen, Moore, & Mohamedally, 2012; Over & Carpenter, 2013; see Hoehl et al., 2019 for review). Seemingly inefficient actions can also indicate that the actor’s goal was simply to perform the movements for their own sake, such as in dance (Schachner & Carey, 2013) or were intended as communicative gestures (Royka et al., 2022), instead of reaching for or manipulating other objects.

However, children may nonetheless believe that violations of efficiency are *characteristic* of play—even relatively straightforward, goal-directed exploratory play. Consistent with this, there is some evidence that children are especially likely to imitate seemingly unnecessary means to an end in playful contexts (Nielsen, Cucchiaro, & Mohamedally, 2012; Schleihauf et al., 2018; see Hoehl et al., 2019 for discussion). Here we ask whether, in the absence of any other discriminative cues, and given otherwise neutral behaviors (collecting sticks, retrieving a box, pushing buttons), children use a violation of efficient, rational action to decide who is playing.

### Methods

#### Participants.

In Experiment 1, we tested 24 children (13 females; mean age: 4.95 years, *SD* = 1.24, range = 37–83 months). The hypothesis that children would identify the inefficient actor as playing was specified ahead of data collection, but not formally pre-registered. Given our directional prediction, we chose this sample size to yield 80% power to detect a moderately large effect (Cohen’s *g* = 0.25). Four additional participants were excluded for ambiguous responses (*n* = 2) or not being fluent in English (*n* = 2).

All children in this and the following experiments were recruited and tested between June 2019 and February 2020 from an urban children’s museum in the United States. Each child participated in exactly one experiment. Parents provided informed consent, and children received stickers for their participation. Although we did not collect participants’ demographic information, participants reflected a range of ethnicities and socioeconomic backgrounds from the museum visitors: 70% White, 3% Black, 9% Asian, 7% other races, 11% two or more races, 10% Hispanic or Latino (any race) and with about 30% of attendees visiting on days when there is free or discounted admission (Boston Children’s Museum, 2023).

#### Materials and procedure.

Participants were tested individually in a quiet room. The experimenter explained that they would watch three stories, each involving two child characters attempting the same task. She explained that “one of the children will just do what they’re supposed to do” and the other child “will play”. Participants were told to guess who was playing.

**Figure 1.**
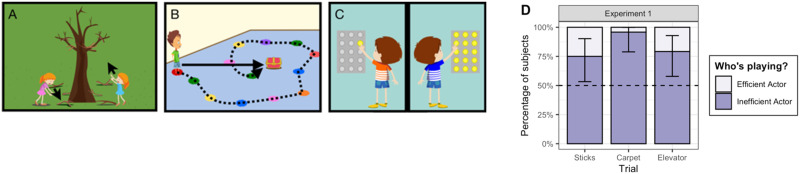
**Stimuli used in Experiment 1.** Each story showed two characters either efficiently or inefficiently completing a task. (A) Asked to retrieve sticks to make a fire, one character reaches efficiently for sticks on the ground and the other reaches inefficiently for sticks on a tree. (B) Asked to retrieve a key from the red box, one character runs efficiently in a straight line and the other hops inefficiently in a spiral. (C) When taking the elevator to go home, one character presses just one button and the other presses all the buttons. (D) Children preferentially identified the inefficient actor as the one who was playing. Error bars show 95% bootstrapped confidence interval on the average response.

The stories were presented on a laptop computer in a fixed order (sticks, key, then elevator; see Figure 1A–C). On each trial, one character acted efficiently and the other acted inefficiently towards the goal. Each character’s behavior was shown sequentially, one on the left and one on the right side of the screen, with order and side randomized at each trial. Trials 1 and 2 were animated: On Trial 1, characters retrieved sticks for a campfire. The efficient actor bent down to get sticks easily accessible on the ground; the inefficient actor jumped and tried to get sticks that were out of reach on a tree. On Trial 2, characters retrieved a key from a box in the center of a room. The efficient actor ran straight to the box; the inefficient actor hopped in a circle. On Trial 3, characters had to ride an elevator to the 14th floor. The efficient character’s panel had one lit button and the inefficient character’s panel had fifteen lit buttons. This trial used only a static image, removing direct cues to action efficiency but previous work suggests that preschooler can infer actions on objects from static images of their end state (Jacobs et al., 2021; Lopez-Brau et al., 2022; Pelz et al., 2020; Pesowski et al., 2020). On each trial, after the child saw both events, the experimenter asked, “Who was playing?”. Results (whether children selected the efficient (0) or inefficient (1) action) were coded live by the experimenter.

### Results

Figure 1D shows the proportion of children choosing the inefficient actor on each trial. More than half the children chose the inefficient actor at ceiling (14/24; 58%) and all but one of the remaining children selected the inefficient actor on two out of three trials (*n* = 9 or 38%). We predicted trial-level responses using a mixed effects logistic regression model with random by-subject intercepts. The null model (syntax: choice ∼ [1|subject]) indicates that children chose the inefficient actor more often than chance of 50% (*β*_intercept_ = 1.84, OR = 6.27, 95% CI: 2.35–16.7, *p* < .001). Adding a fixed effect of trial did not significantly improve the null model (*χ*^2^(2) = 5.5, *p* = 0.06). Adding a fixed effect of age (in months) did improve model fit (*χ*^2^(1) = 4.74, *p* = .03; *β*_age_ = 0.06, OR = 1.06, 95% CI: 1.00–1.12), however, this age trend did not hold after removing the single outlier participant who always chose the efficient actor.

### Discussion

The results suggest that, in these contexts, violating principles of rational action contributed to children’s tendency to attribute a behavior as play. While some of the target behaviors—jumping in the air, running in circles and pushing many buttons—may be actions that are familiar to children as play, it is also (and presumably more commonly) the case that picking up sticks from the ground, running towards a goal, and pushing buttons are also familiar actions in play. Thus, children could not have identified playful behaviors using only surface features of the actions.

As noted, there is no one-to-one mapping between inefficient actions and play. Given a different forced-choice context, children might have been equally likely to identify the inefficient actor as “naughty” or “silly” or in pursuit of an opaque, non-obvious goal. The key point for the current purposes is simply that in the absence of any other cues, children relied on the distinction between efficient and inefficient actions to distinguish play and non-play behaviors.

However, given the forced choice context in Experiment 1, we cannot be sure whether children identified the inefficient actions as play, identified the efficient actions as not playing, or both. Additionally, the inefficient actions were arguably more salient than the efficient ones (i.e., involving longer trajectories or more activated buttons); children might have chosen the targets because they were visually more interesting rather than because they violated efficiency per se. Thus, Experiment 1 provides only suggestive evidence that children understand play as the willingness to violate efficiency and adopt unnecessary costs. Note also that this was a preliminary investigation, and the experimenter was present and not blind to condition. Although she tried to maintain a neutral expression and gaze throughout, future work should replicate the design with appropriate blinding. For the current purposes however, Experiment 1 was intended primarily as a proof of concept, informative mainly in the context of the subsequent experiments. A stronger test of the hypothesis that play is characterized by manipulated utility functions would be whether children *themselves* engage in unnecessarily costly actions in play. We turn to this question in the experiments that follow.

## EXPERIMENT 2

In Experiment 2, we used a retrieval task (analogous to the picking up sticks and retrieving the key stories from Experiment 1) to look at whether children themselves would respect principles of rational action in non-playful contexts but violate them in play. Children were placed in identical environments with identical targets and given either functional or play instructions (“*Could you help me? Maybe you could try to get [the target]*”; “*Could you play over there? Maybe you could play a game to get [the target]*”). We used a within-subjects design to compare children’s choices in both contexts. We predicted that children would retrieve the targets efficiently in the functional context but perform unnecessarily costly actions in the play context. This experiment was pre-registered on the Open Science Framework (https://doi.org/10.17605/OSF.IO/6VQZ5).

Critically, however, even in the playful contexts we did not expect children’s play to be characterized by random or haphazard actions. Rather, we believed that children would act efficiently with respect to their self-imposed costly actions. That is, we expected that children would perform “conditionally efficient” actions: actions that were efficient with respect to the playful goal of adopting a novel, manipulated utility function. To ensure that we could code the efficiency of children’s actions reliably, we intentionally included costly affordances in the environment that children could readily exploit.

We did not include three-year-olds in this study. The affordances we included (the pencils on the wall and the dots around the spiral) were just out of reach for four to five-year-olds. For shorter three-year-olds, exploiting these affordances might have been much more difficult or impossible; thus, if younger children played differently than older children, we would not know if it was an effect of age or the ways these affordances interacted with children’s age. We return to younger preschoolers again in Experiment 3 (using a design that eliminated height as a factor).

### Methods

#### Participants.

We aimed to test 40 participants to yield 80% power to detect a medium effect of condition (odds ratio of 2.5). Our final analyses included 38 children (24 females; mean age = 5.02 years, *SD* = 0.52, range = 49–69 months). Sixteen additional children participated but were replaced: Many (*n* = 10) were excluded on a single day due to a video camera failure; six others were excluded over the course of the experiment due to experimenter error (*n* = 2), incomplete participation (*n* = 3), or parent interference (*n* = 1). After coding the videos, we excluded two additional children for incomplete video clips and the pandemic prevented us from replacing these participants. Participants were randomly assigned to one of four counterbalanced orders (Play or Instrumental first, and Pencils or Stickers task first).

#### Materials.

The Pencils and Stickers tasks took place in two adjacent rooms. The Pencils room was also used for a ten-minute distractor task (part of an unrelated experiment) in which children answered questions about short stories.

**Figure 2.**
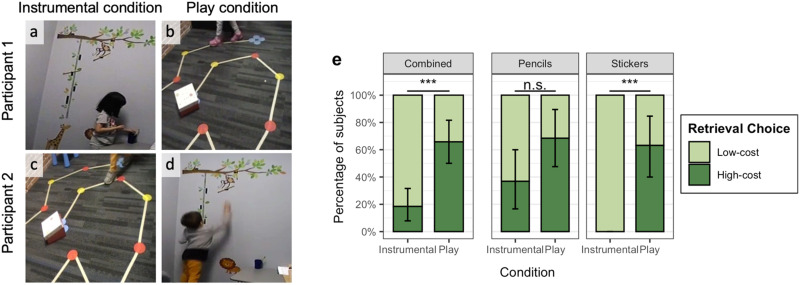
**Experiment 2.** Participants completed one Instrumental trial and one Play trial. Half the participants were assigned to the Instrumental pencils task and Play stickers task; the other half were assigned to the Instrumental stickers task and Play pencils task. Here, one child (A) efficiently retrieved pencils but later (B) took the high cost, inefficient action of walking in a spiral for the stickers. Another child (C) efficiently walked in a straight line to the stickers but then (D) took the high cost, inefficient action of jumping up to get the out of reach pencils. To best illustrate the tasks for each condition, here we presented two children who both got the Instrumental condition first and then the Play condition. However, order was counterbalanced throughout such that half the children got the instrumental condition first and half the Play condition first. (E) Our pre-registered analysis (combining tasks) found that children take low-cost, efficient actions (going straight to the target) in Instrumental conditions and high-cost, inefficient actions (taking unnecessary detours, jumping for out-of reach objects) in Play conditions. Post-hoc analyses found that in both tasks, numerically more children take high-cost actions during Play, but the effect was only significant within the Stickers task. Error bars show bootstrapped 95% confidence intervals.

The room used for the pencil task had a colorful wall decal showing a large horizontal branch (about 6′ off the ground) and a vertically hanging vine (see Figure 2A). Pencils were attached to this wall decal using Velcro at three different heights: 48″ (taller than participants but within easy reach), 58″ (requires a stretch) and 66″ (requires jumping). Next to the wall decal there was a small desk (22″ tall) containing a cup of pencils within easy reach of the children.

The room used for the Stickers task had a carpet on which we placed colorful dots (diameter 4″) along a zigzag spiral (see Figure 2B). Dots were connected with straight lines of tape. The apparent start of the route was at the door where participants entered the room. A small box of stickers was placed on the dot in the very center of the spiral.

#### Procedure.

Children completed one Play and one Instrumental trial with trial and task order counterbalanced across participants. See Figure 2A–D for a schematic of the study design.

To begin a trial, the experimenter walked to the door of the appropriate room and then pretended to suddenly remember something. For Instrumental trials, the experimenter said, “*Oh, I need [some stickers /a pencil]. There’s [some in that box/one over there]. Could you help me? Maybe you could try to [get that box of stickers/get a pencil].*” For Play trials, the experimenter said, “*I have to do some paperwork with your parent. Could you go play over there? Maybe you could play a game where you try to [get that box of stickers / get a pencil]*.”

We took several steps to minimize the chance that adult behavior could influence the children during the tasks. The parent remained in the hallway with the door cracked open but out of sight of the child throughout both tasks. During the pencils task, the experimenter sat in the back of the room at an adult desk, with her head down, completing the paperwork until the child returned with pencils. During the stickers task, the experimenter remained outside of the room with the parent.

Our primary dependent variable was whether children performed any high-cost action as part of their first retrieval attempt. On the Pencils task, we coded low-cost behavior as retrieving a pencil from the cup and high-cost behavior as retrieving a pencil from the wall. On the Stickers task, we coded low-cost behavior as only walking or running straight to the box of stickers, and coded high-cost behavior as taking unnecessary detours (e.g. following the winding path) or performing other self-handicapping behaviors (e.g. hopping, tiptoeing, etc.). Coding decisions were made live by the experimenter and verified from video by a second coder naive to condition, with 100% inter-rater agreement on the binary judgment of high-cost/low-cost behavior. See https://osf.io/2tde5/ for video examples of the task.

Additionally, to see if children who chose the more costly actions in the Play condition acted randomly or were “conditionally efficient” with respect to the goal they chose, coders naive to hypotheses and task conditions were asked to annotate children’s behavior. For children who chose to retrieve a pencil from the wall, a single coder, blind to hypotheses and conditions, judged if children deviated from a direct vertical reach by more than one hand-width. For children who approached the stickers in roundabout paths, a separate coder, also blind to hypotheses and conditions, judged whether children deviated from the spiral path on the ground by more than one footstep.

### Results

All children achieved the retrieval goal. The pre-registered analysis was the within-participant analysis, collapsing across tasks (pencils and stickers) looking at the difference between children’s behavior in the Instrumental and Play conditions. As predicted, children’s behavior differed in the two conditions (OR = 7.00, 95% CI: 2.09–36.65; exact McNemar’s *p* < .001). The majority of participants performed low-cost actions in the Instrumental condition (*n* = 31 of 38 children or 82%, 95% CI: 68–92%) and high-cost actions in the Play condition (*n* = 25 or 66%, 95% CI: 50–79%; see Figure 2E).

We also ran three post-hoc analyses. First, we compared children’s behavior across conditions within each task. In both the pencils and stickers tasks, children performed high-cost actions numerically more often when the task was presented in the Play condition than the Instrumental condition but this effect was only statistically significant in the stickers task (high-cost actions stickers task: 12/19 children (63%) in play vs. 0/19 (0%) when instrumental; Fisher’s exact *p* < .001; high-cost actions pencils task: 13/19 children (68%) in play vs. 7/19 (37%) instrumental, Fisher’s exact *p* = .10; see Figure 2F).

Second, we looked at whether there were any effects of order on children’s performance. There were none. On Play trials, children were likely to act inefficiently whether the Play trial was presented first (*n* = 14/19 children or 74%) or last (*n* = 11/19 or 58%, Fisher’s exact *p* = .49). On Instrumental trials, children were likely to act efficiently when Instrumental trials were presented first (*n* = 17 of 19) or last (*n* = 14 of 19, Fisher’s exact *p* = .4). The absence of order effects was perhaps unsurprising given that the Play and Instrumental tasks took place in different rooms and the intervening ten-minute distractor task was likely to mitigate against carry over effects. However, to ensure that results were not due to children reacting primarily to the contrast in task instructions, we also ran a between-participants analysis of children’s behavior looking just at performance on the first trial. Children performed significantly more high-cost actions in the Play condition than the Instrumental condition when both were the first trial (high-cost actions Play: 14/19 children (74%), high-cost actions Instrumental: 2/19 (11%); binomial *p* < .001).

Finally, we asked whether children in the Play condition performed random, haphazard actions or acted efficiently with respect to the higher cost actions they’d undertaken. Of the 13 children who reached for a pencil on the wall, every child reached straight up for their target pencil; no child ever veered from this direct reach by more than one hand-width. Similarly, of the 12 children who did not run straight to the box of stickers, 11 stuck to the spiral path. Only one child ever veered more than one foot-width away from the spiral path (and she only did so for a few steps before returning to the spiral path—which she then completed for two full rounds before taking a sticker). Thus, relative to children in the Instrumental condition, children at play did incur unnecessary costs—but not by acting randomly. Instead, they appear to subvert the real-world utilities of the task environment, acting efficiently conditional on a manipulated utility function which respects additional constraints.

To enable this kind of coding, we intentionally built in costly affordances that children could choose to exploit. However, consistent with the idea that children spontaneously manipulate utility functions in play, some children also adopted idiosyncratic costs of their own. For instance, in the Play pencils task, one child used the first pencil as a tool to swat at another pencil, and in the Play stickers task, six children went around the spiral twice before retrieving the stickers, one child went around five times (and at one point retrieved the stickers but returned them to the box before going another round!), and one child walked around the spiral backwards. In the Instrumental pencils task, 7 of the 19 children reached for the harder to get pencil on the wall, but they all immediately gave the pencil to the experimenter without taking any other actions. We did not observe any spontaneous extra costly behaviors in the Instrumental stickers task (all 19 children headed straight for the stickers).

### Discussion

When acting instrumentally, children acted rationally and took the most efficient routes to their goals. However, even given identical extrinsic targets and environmental contexts, children adopted higher costs during play. These results support our hypothesis that children’s play is characterized by manipulated utility functions in which children take actions that are unnecessarily costly given (only) the functional goals of the task. This tendency manifested across two quite different task contexts and encompassed a range of different behaviors (reaching and jumping in the pencil task, walking in a spiral in the sticker task), including some spontaneous behaviors (e.g., using one pencil to get another, repeating the spiral, walking backwards around the spiral) not particularly predicted by us as the experimenters. As expected however, when children were told to play, they did not act randomly or haphazardly. They adopted unnecessary constraints but then acted efficiently with respect to those constraints.

In this experiment, the particular actions children generated in play were (as intended) influenced by the affordances of the environment: the pencils stuck to the decal on the wall and the spiral on the ground. Superficial features of these environmental scaffolds may also account for observed task differences, such as the higher occurrence of some playful high-cost actions in the Instrumental condition of the Pencils task (compared to no high-cost actions in the Stickers task. For example, children might have found the pencils on the wall to be more novel or interesting than the patterns on a rug and thus they may have been more interested in jumping up the wall than following a spiral pattern. Alternatively, children might have found obtaining a sticker for themselves more rewarding than obtaining a pencil for someone else and thus they were more inclined to act efficiently. Critically, however, both tasks yielded a difference in performance in the Instrumental and Play conditions, and similar rates of high-cost actions on the Play trials, consistent with our proposal that adopting alternative utility functions may be characteristic of play.

## EXPERIMENT 3

Experiment 2 looked at children’s behavior in instrumental and playful retrieval tasks and found that children at play violated principles of rational action. However, one caveat is that children in the Instrumental condition were asked to retrieve an object that the experimenter needed (the pencil or the sticker box). Although some children may have understood that the stickers were meant for their benefit, the pencil was clearly for the experimenter’s; arguably, children might act more efficiently when acting on behalf of others than when acting for themselves. In Experiment 3, we control for this possibility by asking children in the Instrumental condition to retrieve objects needed for outcomes desirable for themselves.

Additionally, Experiment 2 focused on rational action but as described in the Introduction, accounts of rational exploratory play also presume that children explore efficiently to gain information. To test the generalizability of children’s tendency to take on unnecessarily costly actions during play, in Experiment 3, we asked whether children show a similar distinction between instrumental and play behavior using a search task rather than a retrieval task.

We set up tasks in which a target was equally likely to be found in either a small or large search space (i.e., among one or twelve drawers; or on a toy with two or eight buttons). If children start by searching in the smaller space, their first action will be highly informative: They would either find the target within moments of exploring, or if they fail to find the target in the small search area they will quickly know for certain that the target is in the larger search space. By contrast, if children begin by searching in the larger space, their first actions will be only minimally informative, and unlikely to be fruitful: They will have to search extensively (through many of the drawers/buttons) before either finding the target or knowing for certain that the target is in the smaller space.

Thus, if children are sensitive to the size of the search space, the rational decision is to first search in the smaller space. To our knowledge, no research has previously demonstrated that preschoolers are indeed sensitive to the size of search spaces, so this question is of interest in itself. (See Ruggeri et al., 2017 for work on preschoolers’ sensitivity to the size of hypothesis space in question-asking tasks.) However, our primary question was not whether children would search rationally in the Instrumental condition but, presuming they do, whether they make different decisions and choose to take on unnecessary costs in play. Secondarily, as in Experiment 2, we expected that even children who violated efficiency in play would not act randomly. We thus looked at whether children who chose the high-cost tasks in the Play conditions nonetheless searched efficiently conditional on the task they had chosen.

As noted, no previous work has looked at whether preschoolers understand that it is easier to search in smaller search spaces than larger ones—let alone whether they understand that if a target is equally likely to be in each location, it is rational to begin with the smaller space. If children fail to understand this, then their tendency to violate rational action (in either condition) might simply reflect a poor understanding of the task. Although a general failure to understand the paradigm cannot, in itself, account for any condition differences that might emerge, we nonetheless thought it was important to assess whether children understood the rational decision in these tasks. Thus, at the end of the experiment, after children indicated where they wanted to start searching in the Play condition but before beginning to search, we asked children where they would search if they “really wanted” to find the target. If children indeed know which space is easier to search—despite selecting the more costly space in play—we can be confident that play involves taking on unnecessary costs. Note, however, that this question refers only to children’s explicit understanding of the rational, instrumental action; it does not require that children have any metacognitive understanding that they violate efficient action in play. (See discussion in the Introduction.)

We ran both an original experiment (Experiment 3A) and a replication (Experiment 3B) in which we extended the study to three-year-olds. This was motivated in part by Experiment 1, in which we found no effect of age on children’s judgment that play was characterized by violations of rational action, and was aided by the fact that in Experiment 3, unlike Experiment 2, the height of the child had no bearing on their ability to perform the tasks. These experiments were pre-registered on the Open Science Framework (Experiment 3A, Experiment 3B) [4–5-year-olds], and Experiment 3B [3-year-olds]).

### Methods

#### Participants.

As in Experiment 2, we aimed to test 40 4–5-year-olds in each of Experiments 3A and 3B to yield 80% power to detect a medium effect of condition (odds ratio of 2.5) in each sample. We targeted a smaller sample of 3-year-olds because a sample of *n* = 30 yields 80% power to both replicate the condition effect obtained in 4–5-year-olds (McNemar’s OR = 4.75) and to detect a large difference between the older and younger samples (binomial test, Cohen’s *h* = 0.8).

In Experiment 3A we tested 40 children (12 female) aged four and five years (mean age: 4.87 years, *SD* = 0.58) randomly assigned to one of two counterbalanced task orders (Boxes or Buttons first). Two additional participants were excluded for incomplete participation (*n* = 1) or technical failure (*n* = 1). In Experiment 3B we tested 40 four- and five-year-olds (mean age = 4.80 years, *SD* = 0.53; 16 female) and 29 three-year-olds (mean age = 3.59 years, *SD* = 0.26; 10 female). Eleven additional participants were excluded for parental interference (*n* = 2), exploring toys before prompts were delivered (*n* = 2), incomplete participation (*n* = 2), technical failure (*n* = 1), not making a clear choice (*n* = 1), or experimenter error (*n* = 3). (We had aimed for 30 three-year-olds but the technical error prevented us from recovering a video file on one participant and the pandemic prevented replacing participants.)

#### Materials.

**Figure 3.**
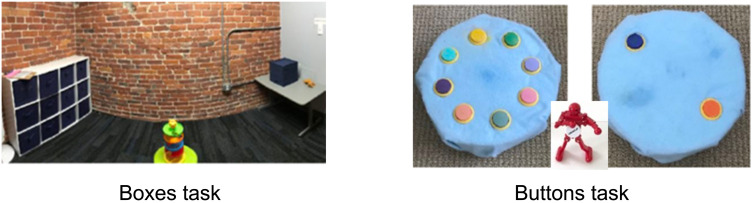
**Experiment 3 materials.** In Experiment 3, a target was equally likely to be found in a smaller or larger search space. On the Boxes task, children searched for a ball in either a shelf with 12 boxes or a shelf with 1 box. On the Buttons task, children searched for a music button on a toy with 8 buttons or a toy with 2 buttons. Children completed one Instrumental trial where the target served a functional goal (to play with a ramp toy in the Boxes task or to make a robot dance in the Buttons task), then one Play trial (“hide and seek”) without any functional goals. Task was counterbalanced between trials.

Figure 3 shows the materials used. In the Boxes task, participants had to search for a plastic ball. The larger search space was a shelf with 12 identical opaque 12″-cubic drawers (in a 4 × 3 array) and the smaller space was a single drawer resting atop a child-sized table. To motivate search in the Instrumental condition we used a colorful ramp toy for the ball to roll down. The experimenter and the children sat facing each other in the center of the room, equidistant from both sets of drawers. In the Buttons task, participants had to search for a button that played music. The larger search space was a round 8-button toy, and the smaller space was a 2-button toy. Both toys were diameter 12″, height 3″ and had colorful buttons evenly spaced around the perimeter. By design, exactly one button on each toy played music. In the Instrumental condition we used a wind-up dancing puppet to motivate search. Varying the ratio of the two options across paradigms allowed us to probe the generality of children’s sensitivity to costs (i.e., whether they merely distinguished one and multiple alternatives or whether they also distinguished between fewer and more alternatives).

The Boxes task was presented identically between Experiments 3A and 3B with accompanying gestures to emphasize uncertainty about which side of the room the ball was in; children were told there was only one ball that could be in either set of drawers (“*in one drawer over there or in one of the twelve drawers over there*”). In fact, the ball was always in the drawer in the smaller search space, so that if children searched efficiently, they would find it immediately. The Buttons task was presented with slight modifications between experiments: In Experiment 3A we told children there was one music button on both toys—which was indeed the case (“*One of these two buttons makes music. One of these eight buttons makes music.*”). In Experiment 3B, to match the Boxes and Buttons tasks, we told children there was only one music button and it could be on either toy (“*Maybe one of these two buttons could play music, or maybe one of these eight buttons could play music.*”). In fact, however, we used the same toys as in Experiment 3A, so children would find the music button on whichever toy they searched.

#### Procedure.

To control for the possibility that children might be more inclined to act efficiently when acting on behalf of others, the Instrumental tasks in Experiment 3 were for the child’s own benefit: Children were asked to find a ball to slide down a ramp toy and find a button to make a robot toy dance. Given the absence of order effects in Experiment 2, the overall complexity of the design, and the fact that the overarching frame was consistent with both goals, we simplified Experiment 3 by eliminating the distractor task and having all participants complete the two trials in a fixed order: Instrumental search, then Play. On the Instrumental trial, the experimenter introduced the ramp/puppet and then announced that she wanted to play with the ramp/make the puppet dance but couldn’t find the ball/didn’t know which button made music. She pointed out that the target might be in either the small search space or the large search space and said: “*Can you help me find the ball/the button that plays music? It might be over there or it might be over there*”. In Experiment 3A, we always introduced the side with the smaller number of options first; in Experiment 3B, we counterbalanced which side was introduced first. We coded the location of children’s first search (small or larger space).

After the child found the target and used it to roll down the ramp/make the puppet dance, the experimenter transitioned to the Play trial by suggesting they play a new game with the other set of materials. The experimenter explained that she was going to hide a target for the child to find (“*We’re going to play a hide-and-seek game with this ball. I’m going to hide it in a drawer, and you can look for it.*” / “*We’re going to play a hide-and-seek game with this music machine. You get to look for the button that plays music.*”; In Experiment 3B to make it clear to three-year-olds that the music button was hidden she said, “*I’m going to make one button play music and you get to find it.*”) She then asked if the child would like to play in the smaller or the larger search space: “*Do you want to play with the drawers over there or the drawers over there? / With this toy or this toy?*”.

After the child made a choice about where to play, but before the experimenter allowed them to go search, the experimenter asked a final question: “*If you really wanted to find the ball/make music, which side/toy is easier? Looking in one drawer over there or twelve drawers over there/The two button toy or the eight button toy? Why?*” This allowed us to compare children’s choice of where to play with their explicit judgment about which game would be easier, before children had explored themselves.

As in Experiment 2, we took several steps to minimize the chance that adult behavior could influence the children during the tasks. The experimenter and child sat equidistant between the drawer locations, and the button toys were placed equidistant from the child. The experimenter maintained a neutral expression throughout and after giving the instructions, kept her head down until the child returned with the ball/located the music button. To avoid parental interference, parents sat in a far corner of the room, reading an instruction sheet reminding them to look down and remain quiet throughout.

We coded whether children chose the low-cost action, searching first in the smaller search space (i.e., 1 drawer or 2-button toy) or the high-cost action, searching first in the larger search space (i.e., 12 drawers or 8-button toy). Coding decisions were made by the experimenter and from video by a second coder naive to condition. Inter-rater agreement was 98%; disagreements were resolved by reviewing the videos together.

Additionally, we coded whether children who chose the high-cost search task in the Play condition nevertheless searched efficiently within that larger search space. One coder blind to hypotheses and condition looked at whether children checked the twelve drawers in sequence or haphazardly; a separate coder judged whether children who played with the eight-button toy pushed the buttons around the circle sequentially or if they pressed the buttons haphazardly.

### Results

**Figure 4.**
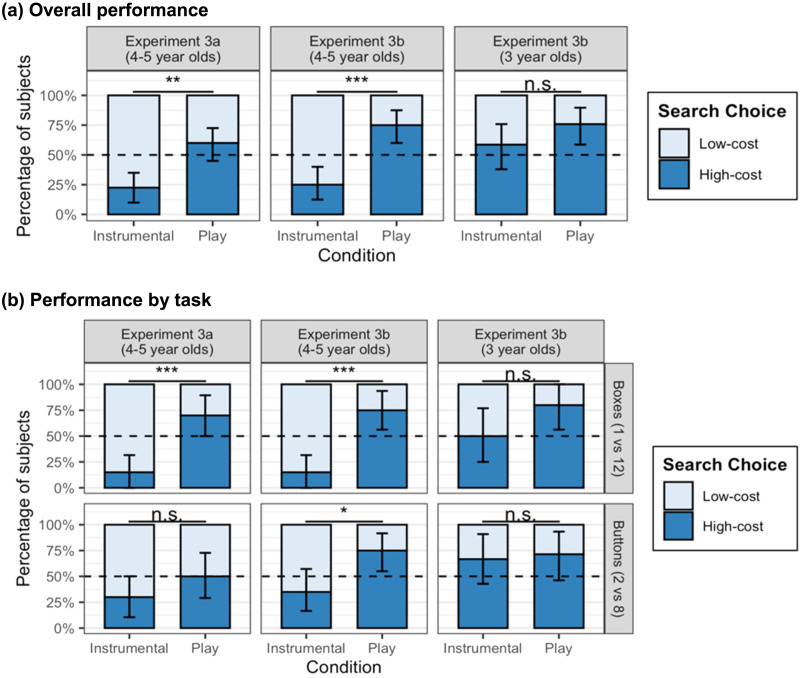
**Experiment 3: Children’s choice of low or high-cost actions in Instrumental and Play conditions.** (A) Our pre-registered analysis, finding that four and five-year-olds take low-cost, efficient actions (searching the smaller search space) in Instrumental conditions but high-cost, inefficient actions (searching the larger search space) in the Play conditions. Three-year-olds showed a similar pattern but the effect was not significant because many three-year-olds chose the larger space even in the Instrumental condition. (B) Post-hoc analyses showing that four and five-year-olds take more high-cost actions during Play than the Instrumental condition in each of the two tasks, although the effect was only significant for the Buttons task in the replication experiment. Three-year-olds a similar, non-significant trend in the Boxes task but no effect in the Buttons task. Error bars show bootstrapped 95% confidence intervals above the mean.

All children searched and found the targets. The pre-registered analysis looked at the results within participants, collapsing across the Boxes and Button tasks (see Figure 4A). As predicted, in both the original experiment (Experiment 3A) and the replication (Experiment 3B), four- and five-year-olds chose to begin searching in the high-cost, larger search space more often on the Play trial (Exp. 3A: *n* = 24/40 or 60%; Exp. 3B: *n* = 30/40 or 75%) than the Instrumental trial (Exp. 3A: *n* = 9/40 (23%), OR = 4.75, 95% CI: 1.6–19.2, McNemar’s *p* = .003; Exp. 3B: *n* = 10/40 (25%), OR = 7.67, 95% CI: 2.3–39.9, *p* < .001). Children’s choices differed from chance responding: On Play trials children chose the larger search space more often than chance (Exp. 3A: binomial *p* = .27; Exp. 3B: *p* = .002). By contrast, on Instrumental trials, children chose the low-cost, smaller search space significantly more often than chance (Exp. 3A: binomial *p* < .001; Exp. 3B: *p* = .002). However, this is not because children failed to understand the task. Most children correctly answered that they would search the smaller space if they “really wanted to” find the target (Exp. 3A: *n* = 29/40 (73%), 95% CI: 58–85%, *p* = .006; Exp. 3B: *n* = 30/40 (75%), 95% CI: 60–88%, *p* = .002), and this was true even among children who chose to play in the high-cost, larger search space (Exp. 3A: *n* = 15/24 (63%), 95% CI: 40–81%, *p* = .03; Exp. 3B: *n* = 21/30 (70%), 95% CI: 51–85%, *p* = .04).

Among the three-year-olds in Experiment 3B, however, condition did not influence children’s preference for the small or large search space (OR = 2.25; 95% CI: 0.63–9.99, McNemar’s *p* = 0.3). Instead, the majority of three-year-olds chose the high-cost, larger search space on both the Instrumental (*n* = 17 of 29, 59%) and Play (*n* = 22/29, 76%) trials. Further, three-year-olds chose at chance when asked where they would search if they “really” wanted to find the target; only 12/29 (41%) correctly identified the smaller option as the easier search task.

We also did a post-hoc analysis looking at children’s behavior within each task, between participants (see Figure 4B). In the Boxes task, four- and five-year-olds chose the low-cost smaller search space more often in the Instrumental condition than in Play (Exp. 3A: *n* = 17/20 vs. 6/20, exact Fisher’s *p* = .001; Exp. 3B: *n* = 17/20 vs. 5/20, *p* < .001). In the Buttons task however, this effect was weaker and only significant in the replication experiment (Exp. 3A: *n* = 14/20 vs. 10/20, *p* = .3; Exp. 3B: *n* = 13/20 vs. 5/20, *p* = .02). While the tasks differed in many respects, it is possible that the greater 1:12 contrast in the Boxes task provided a more compelling cost differential than the 2:8 contrast on the Buttons task.

**Figure 5.**
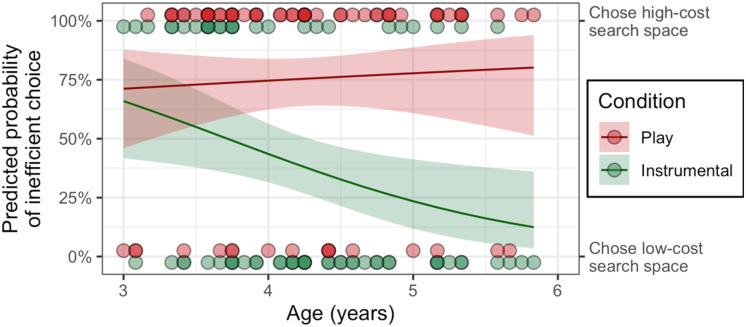
**Age differences in search efficiency in Experiment 3B (*n* = 69).** Older children were increasingly likely to make low-cost, efficient choices in the Instrumental search task (green line). However, in play, children of all ages preferred the larger search space. Each circle represents the choice of one participant and lines show predicted probability of making each choice across the ages tested; shaded regions indicate 95% confidence intervals.

In Experiment 3B we conducted an exploratory analysis to test the effects of age (in months) on selecting the larger search space, using a logistic mixed effects regression with condition and age as predictors and a random by-subject intercept. We found a significant age by condition interaction (*β* = .091, OR = 1.1, 95% CI: 1.00–1.20, *p* = .044). Inspection of simple slopes within trial type found no age effect within Play trials (OR = 1.01, 95% CI: 0.95–1.08). However, there was a significant age effect in the Instrumental trials (OR = 0.93, 95% CI: 0.87–0.99), indicating that a one-month increase in age predicted a 7% lower probability of choosing the high-cost larger search space (see Figure 5). These results held within both the Boxes and Buttons task (see Supplemental Materials for more details).

We also looked at whether children who chose the larger search space in the Play conditions searched efficiently within this space or if they searched randomly or haphazardly. Unfortunately, only after the experiment was complete, it became clear that the camera placement obscured the full search trace on the larger set of drawers in the Play condition (although the children we could observe searched consecutive drawers). The full video data was available for the Buttons task, so we focused on this.

On the Buttons task, we identified 22 children who chose to play on the 8-button toy and who tried at least two buttons (i.e. they did not succeed on the first press). This included eight 3-year-olds and fourteen 4–5-year-olds across Experiments 3A–B. All of the eight 3-year-olds searched the buttons efficiently in order, as did most of the four and five-year-olds. Only four children pushed the buttons in a non-adjacent order: pushing two buttons simultaneously (*n* = 1), repeatedly pressing inert buttons (*n* = 1) or searching in arbitrary sequences (*n* = 2). Thus, even when children chose the more costly, less efficient search space in play, they searched efficiently conditional on that choice.

### Discussion

As in Experiment 2, the results of Experiments 3A and 3B suggest that four- and five-year-olds acted efficiently when exploring for instrumental ends but preferentially incurred unnecessary costs when playing. Note that if the larger search space was simply more salient or exciting and this affected children’s exploratory behavior, four and five-year-olds would have chosen the larger search space in both conditions. Similarly, if children always preferred the fastest route to finding the target and an “easy win”, they would have chosen the smaller search space in both conditions. Instead, the results suggest that four and five-year-old children choose to minimize costs during instrumental search, but selectively choose high-cost actions in play.

Critically however, and as in Experiment 2, even children who chose the high-cost search space went on to search efficiently conditional on their chosen goal: to find the target in that space. Children almost uniformly searched in an organized manner, trying each button once in order. Thus, even when voluntarily incurring unnecessary costs in play, children acted efficiently with respect to the playful utility function.

We also found a developmental effect. Three-year-olds, like older preschoolers, preferred more challenging search problems during play; however, they failed to make efficient search decisions even given instrumental goals. Three-year-olds also chose at chance (*M* = 41%) in identifying the smaller search space when explicitly asked where they would search if they “really wanted” to find the target. As noted in the Introduction, the current study was designed to look at whether children deliberately took on unnecessary costs during exploratory play; since the phenomena had not been established, the study was not designed to look at factors that might affect changes over development. Nonetheless, we can speculate on several, not mutually exclusive, explanations for the age effect.

First, three-year-olds might have failed to represent the size of the search space as a relevant variable at all; they may have chosen the larger search space simply because it was more exciting and failed to consider the implications for efficient action. Alternatively, three-year-olds might have recognized the relevance of the size of the search space for decision-making but been unable to compare and represent the distinction in costs between the two alternatives. Changes in children’s numerical cognition affect their behavior in other tasks involving costs and rewards (e.g., their preference for equal versus merit based sharing; Jara-Ettinger et al., 2016); developments in children’s ability to use numbers as a basis for comparison might have similarly affected children’s performance in this task. In this vein, it is interesting that three-year-olds’ performance was somewhat better in the task with the stronger numerical contrast (the 1 vs. 12 Boxes task vs. the 2 vs. 8 Buttons task).

Alternatively, children might have represented both the relevance of the size of the search space and the distinction in costs but failed to use this distinction as a basis for proactive planning. They may have failed to integrate all the relevant information in time to make a decision—or the larger spaces may simply have been so enticing that three-year-olds could not inhibit the desire to search there.

Note however, that when searching within a given space, three-year-olds, like older children, acted efficiently, checking buttons in order and rarely repeating actions. Thus, it is not the case that three-year-olds fail to understand efficient search altogether. This leaves open another intriguing possibility. It is possible that three-year-olds value efficiency less—or value play more—than the older participants. That is, even when given a putatively functional goal, three-year-olds might always be more likely than older children to choose to play.

Finally, as discussed, we believe these experiments are the first to show that preschoolers are sensitive to the size of a search space in goal-directed exploration and rationally prefer to search first in smaller spaces. This behavior generalized across two quite different contexts (searching for objects in chests of drawers and functional buttons on toys) and contrast ratios (1 vs. 12 and 2 vs. 8). The results suggest four and five-year-olds can anticipate and compare the relative costs of exploration and proactively select easier search problems.

## GENERAL DISCUSSION

Collectively, these results suggest that children’s exploratory play is characterized by apparent violations of principles of rational action. Across three studies, preschoolers used violations of rational action to decide when others were playing (Experiment 1), and to play themselves, voluntarily incurring unnecessary costs in both retrieval (Experiment 2) and search (Experiment 3) tasks. The tendency to incur unnecessary costs in play was not due to children’s failure to understand the possibility of more efficient actions: Four and five-year-olds acted efficiently in instrumental tasks even though the environments were matched across conditions. Moreover, once children decided to take unnecessarily costly actions in play (e.g., jumping for an out-of-reach target or searching in larger search spaces), children as young as three behaved efficiently with respect to these choices (jumping straight up; searching adjacent spaces sequentially). We suggest these results are consistent with the proposal that children’s play is not only boundedly rational (limited by information processing constraints; Simon, 1955) and resource rational (rational with respect to estimates of those processing constraints; Bhui et al., 2021; Lieder & Griffiths, 2020; Ma & Woodford, 2020) but also *conditionally rational*: rational with respect to the child’s self-generated utility functions.

Of course, humans are not the only species that engage in unnecessarily costly behaviors during play (Petrů et al., 2009; Spinka et al., 2001). However, the kinds of constraints and variability that non-human animals incorporate in their play are restricted and closely related to the species’ behavioral and locomotor niche (e.g. somersault play is observed in Patas monkeys, which spend their time mostly in trees, but not Diana monkeys, which spend time running on the ground; Petrů et al., 2009). What’s special about human cognition may not be the mere possibility of manipulating normal utilities but the flexibility with which we can do so.

In these experiments, we deliberately introduced environments that invited specific forms of play (e.g., a spiral path; pencils stuck to a tree on a wall). This made it easy not only to distinguish functional and playful behavior but also to distinguish efficient and random behavior during play (e.g., sticking close to the spiral path or vertical line of the wall versus more haphazard behavior). However, although the environmental affordances offered ready-to-hand constraints, they alone cannot account for children’s behavior since children ignored these cues in functional contexts (e.g., walking straight to the stickers; retrieving the pencil from the cup). The general idea that children’s behavior, even in play, is rational conditional on the self-imposed constraints they have established is consistent with other work on the ways that children respect constraints even within imaginary contexts. Thus for instance, preschoolers mop up the pretend pig who got muddy, not the one who stayed clean, and the pretend tea precisely where it spilled, not anywhere else (Harris & Kavanaugh, 1993; see also Gendler, 2000; Lewis, 1978; Weisberg & Bloom, 2009; see Harris, 2021 for review and discussion).

Note however, that we do not have direct access to children’s subjective utility function, and children may have imagined additional constraints beyond those that were clear given the affordances we provided (e.g. perhaps children decided to search especially slowly or quickly, or push the buttons harder than necessary). And it is of course possible that in addition to searching the larger search space in play, children could have generated additional constraints (e.g., open every other drawer first) that would have looked inefficient to us (rather than, as we observed here, conditionally efficient given the self-imposed goal). As it happens, we did not see observable cues to additional constraints here; however, future work could further investigate the nature of children’s self-generated playful utility functions by asking children to provide verbal reports of their plans or by measuring play in environments with more fine-grained parameters that children may selectively manipulate.

In these experiments, we used explicit language about play throughout. In principle therefore, our results might bear more on children’s understanding of the meanings of the word *play*, or *game*, than on play itself. Games—a canonical form of play—are indeed characterized by manipulated utility functions: arbitrary rewards achieved at unnecessary costs. So, perhaps children’s imposition of constraints was due specifically to their understanding of what it meant to “play a game”. However, the constraints in games are pre-established and conventionalized; whereas here children spontaneously adopted ad hoc constraints for each specific task. And although possible, we don’t think there are strong grounds for believing children’s behavior would differ between the instructions “Can you play *a game* in here to get the stickers/pencil” versus “Can you *play* in here and get the stickers” (and similarly, for “which toy do you want to play a hide and seek game with?” versus “which toy do you want to play with?”) By contrast, if we had explicitly told children, “Do anything you want in here”, we suspect that children might have played in many different ways beyond what we observed here (e.g., sticking stickers throughout the room, drawing monkeys on the tree, etc.). Such behavior would be richer and more idiosyncratic—and correspondingly harder to code—but would also involve setting costly goals. That is, we believe the particular behaviors we observed were due to the opportunities the environment afforded for play rather than the instructions to “go play” per se.

In this study, we constrained the extrinsic rewards by assigning target goals—and these goals were so easy to achieve that there were no obvious ways that children could relax the constraints to make the tasks any easier. Perhaps unsurprisingly therefore, in our tasks, children modified their behaviors exclusively by taking on unnecessarily costly actions. Arguably then, children’s play may be characterized specifically by making an easy task more challenging rather than the ability to manipulate their utility function broadly. Clearly however, children do not always take more pleasure in more challenging tasks. (Few children would opt to walk twice as far to the school bus or search twice as many rooms for their shoes just for the pleasure of it.) Alternatively then, children’s pleasure may stem not from merely imposing higher costs but from their ability to choose the costs themselves. Indeed, when children are asked to explain what makes an activity playful, they often point to the importance of autonomy and choice (Goodhall & Atkinson, 2019). Even in playing games, which specify not only the goals but also the costs and constraints to be followed, children may take pleasure in the ability to choose and plan their actions, rather than following standard behavioral scripts or acting in the most obvious ways possible. Future work may investigate how the degrees of freedom for acting and planning shape children’s decisions about which games to play and their sense of fun.

We suspect however, that we might also have induced a sense of play by allowing children to work for arbitrary rewards. We might, for instance, have compared children’s responses to functional instructions: “If you go down that hallway, you can get the part we need for this balloop toy to work” versus playful ones like, “If you go down that hallway, you can get 30 balloop points”. We predict that children would run faster for the arbitrary reward of “balloop points’ than the functional end. Similarly, if we told children “You can put the pencils in this box here" (a functional end) or “you can put the pencils in this box with a hole in the bottom over here” (a playful end) we predict that children would opt to “clean up” the pencils in the bottomless box. Such thought experiments suggest that it’s not just the willingness to incur unnecessary costs but the ability to manipulate utilities– costs or rewards or both—that children find pleasurable. Future research might look at the extent to which children in play vary their utilities broadly (e.g., by relaxing instead of imposing constraints, or by varying the reward function).

As noted, the value of setting your own goals has become increasingly clear in the fields of AI, machine learning, and robotics (e.g., Chitnis et al., 2020; Florensa et al., 2018; Gottlieb et al., 2013; Haber et al., 2018; Kaelbling, 1993; Lynch et al., 2020; Sukhbaatar et al., 2017). Unlike agents hardwired or trained to perform pre-specified tasks, agents who set their own goals in pursuit of intrinsic rewards can learn flexibly even when extrinsic rewards are sparse (see e.g., Colas et al., 2020; Linke et al., 2020; Oudeyer et al., 2007 for reviews and discussion).

However, the current results suggest that neither extrinsic nor intrinsic rewards as traditionally conceived (e.g., rewards tied to learning) adequately account for distinctively human play, even in very simple contexts like those in the current study. We suggest that the distinctively human ability to manipulate our own utilities allows us unusual flexibility in setting new goals and creating new problems for ourselves. Rewards—even in the form of information gain—are often sparse. To the degree that we can generate reward for ourselves by creating new problems and planning and thinking within those constraints, we may be able to think of plans and ideas we wouldn’t have otherwise.

We believe these results are consistent with the idea that humans not only have a remarkably flexible ability to reshape our utility functions but also find it intrinsically rewarding to do so. When children explored for instrumental ends, their utilities were determined by the most efficient way to achieve the target goals. But when children were told to play, they seemed to interpret this as an invitation to manipulate the normal utility function. Indeed, given that this difference in utilities was all that distinguished the tasks, the mere ability to manipulate typical utility functions apparently sufficed to make the task count as play.

Thus, the reward value associated with play might not be tied to *learning* per se but to *thinking*. Inventing problems we don’t (actually) have might be a way of generating solutions we don’t (currently) have. On this account, play is not (only) a means of gaining information. Liberated from any practical goals—even the goal of learning—play may be a means of increasing innovation. Our capacity to invent and solve small problems, and to find it rewarding, may help human learners solve a big problem: the problem of how to generate new ideas and plans in an infinite search space.

Still, why invent arbitrary utilities to generate novel ideas and plans, rather than simply explore in ways that are consistent with real world utilities and immediately likely to improve our policies and increase our knowledge of the world? One possibility is that play solves a meta exploration/exploitation problem: We can exploit our existing knowledge about valuable ways to explore (e.g., by acting efficiently to reduce uncertainty and maximize expected information gain) but we can also explore alternative ways to explore. A sure way to generate novel exploration policies is to manipulate typical utility functions by adopting unnecessary costs and trying to achieve arbitrary rewards. The world is full of unknown unknowns: if we only explored in ways consistent with expected information gain, we would miss the chance to learn the unexpected.

At this point, these ideas about the larger role of play remain speculative. But the function of play—the most characteristic behavior of our most powerful learners—has remained elusive despite decades of research. We believe there may be something to be gained by taking the seeming “uselessness” of play seriously. We hope this work contributes to asking new questions about its value.

## ACKNOWLEDGMENTS

We are grateful to the families who participated in this research. We thank Lucy Fu and Olivia Valle for assisting with subject recruitment and data collection and Sophia Diggs-Galligan, Sofia Riskin, Nicole Coates, Jessica Chomik, Maya Taliaferro for help with data annotation, and all members of the Early Childhood Cognition Lab for their thoughtful feedback.

## AUTHOR CONTRIBUTIONS

Junyi Chu: Conceptualization; Formal analysis; Writing—Original draft. Laura E. Schulz: Conceptualization; Writing—Review & editing.

## FUNDING INFORMATION

This research did not receive any specific grant from funding agencies in the public, commercial, or not-for-profit sectors.

## DATA AVAILABILITY STATEMENT

All data, analysis code, materials, and pre-registrations are available at https://doi.org/10.17605/OSF.IO/2TDE5. The primary video data are not publicly available because they include videos of children, however, a few example videos are included in the repository with explicit parental consent.
