# Supplementary material for: Not Playing by the Rules: Exploratory Play, Rational Action, and Efficient Search
Source: Open Mind (Camb). 2023 Jun 15;7:294–317. doi: 10.1162/opmi_a_00076 (PMC10320825; doi:10.1162/opmi_a_00076)
Supplement: Supplementary file 1 [file opmi-07-294-s001.pdf]

Supplemental Materials for
Not playing by the rules: Exploratory play, rational action, and efficient search

In the main manuscript, Experiment 3b, we reported an exploratory analysis of the effect of age on children's selection of the smaller or larger search space (Figure 5, main manuscript). Here we conduct an exploratory reanalysis of Experiment 3b, asking if the effect of age and condition might differ across both tasks (Boxes and Buttons).

We fit a logistic mixed effects regression model with condition (Instrumental or Play), age (in months), and task (Boxes or Buttons) as predictors. We included all two- and three-way interactions, as well as a random by-subject intercept. Note that compared to the original model reported in the manuscript, which excluded the task variable, this expanded model did not explain any significant additional variance (^{2}(4) = 2.40, p = 0.66).

Investigating simple slopes of age within each task and condition combination, we found that in the Play conditions, children of all ages preferred to choose the high-cost larger search space (see Figure S1). There was no age effect on the Play conditions for either task (Boxes: β = .019, OR = 1.02, 95% CI:0.92-1.13, p = .71; Buttons: β = .014, OR = 1.01, 95% CI:0.94-1.10, p = .74).

In contrast, in the Instrumental condition, older children were increasingly likely to choose the low-cost smaller search space in both tasks, although these estimates did not differ significantly from zero (Boxes: β = -.083, OR = 0.92, 95% CI:0.84-1.01, p = .08; Buttons: β = -.07, OR = 0.93, 95% CI: 0.85-1.02, p = .14).

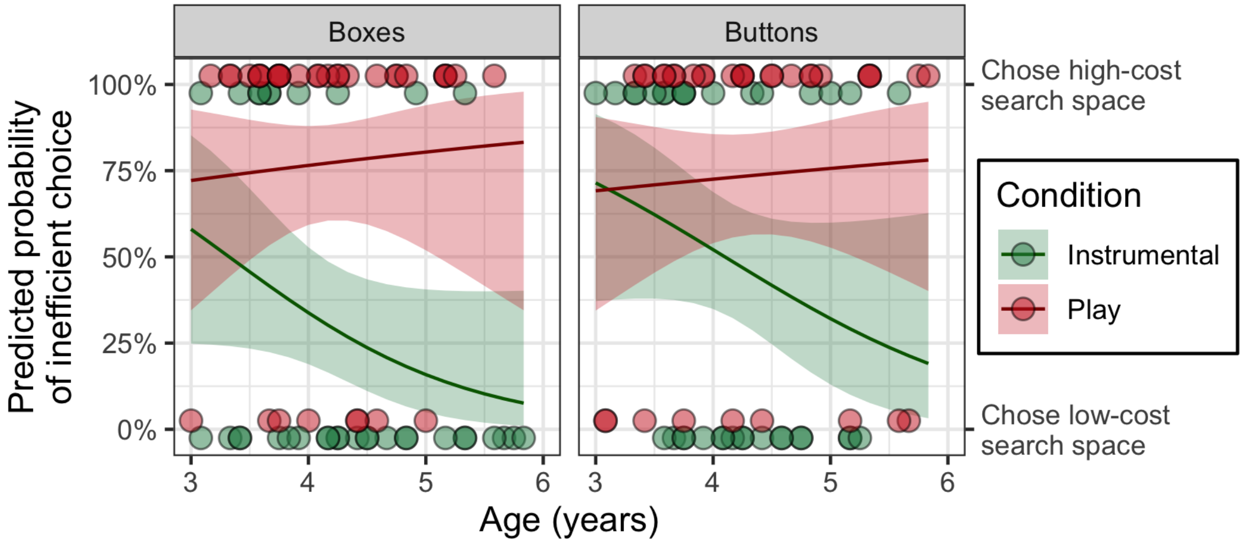

Figure S1. Age differences in search efficiency in Experiment 3b (N=69). In both tasks, older children were increasingly likely to make low-cost, efficient choices in the Instrumental search task (green line). However, in play, children of all ages preferred the larger search space. Each circle represents the choice of one participant and lines show predicted probability of making each choice across the ages tested; shaded regions indicate 95% confidence intervals on best-fit logistic regression estimates.
